# Schell on Operation on Fistula in Ano with the Elastic Ligature

**Published:** 1874-07-01

**Authors:** 


SCHELL ON OPERATION ON FISTULA IN ANO WITH THE ELASTIC LIGATURE.—Dr. H. S. Schell describes, in the *Philadelphia Medical Times* for February 28, a case in which he operated on fistula in ano by the elastic ligature. The patient was a man, Michael D., aged thirty-six. The fistula, which had existed for several years, was small, opening in the skin about half an inch, from the verge of the anus, and within the bowel somewhat over an inch from its termination, embracing but little more than the external sphincter ani. The ligature was drawn through the fistula by means of an ordinary eyed probe, brought down outside the rectum, and tied pretty tightly.

An opium suppository was prescribed, to be used in case any pain followed the constriction of the parts. The patient, however, stated that he had no pain at all, and went about the ward as long as he remained in it, assisting the nurse in the care of the other patients. The ligature came away at stool on the fourth day, leaving a granulated wound, to which no further attention was paid, except as to cleanliness, and which healed in the usual manner.

Dr. Schell remarks that the advantages to be derived from the employment of the elastic ligature in this operation appear to be the following: 1. There is no pain. 2. Hæmorrhage is entirely avoided. 3. There is no need of confinement to bed. 4. The bowels may be left to their ordinary regular habits.

The best ligature is composed of three strands of caoutchouc, somewhat compressed within a plaited envelope of white silk into a round cord, and has the strength of an ordinary ligature. The quality of the ligature should be tested before use, as it grows brittle with age.

